# Spatial Multiomics of Lipids, N-Glycans, and Tryptic Peptides on a Single FFPE Tissue Section

**DOI:** 10.1021/acs.jproteome.2c00601

**Published:** 2022-10-19

**Authors:** Vanna Denti, Giulia Capitoli, Isabella Piga, Francesca Clerici, Lisa Pagani, Lucrezia Criscuolo, Greta Bindi, Lucrezia Principi, Clizia Chinello, Giuseppe Paglia, Fulvio Magni, Andrew Smith

**Affiliations:** † Department of Medicine and Surgery, Proteomics and Metabolomics Unit, University of Milano-Bicocca, 20854 Vedano al Lambro, Italy; ‡ Bicocca Bioinformatics Biostatistics and Bioimaging B4 Center, School of Medicine and Surgery, University of Milano-Bicocca, 20900 Monza, Italy

**Keywords:** MALDI-MS imaging, lipidomics, N-glycomics, proteomics, spatial proteomics, renal cancer, tumor, multiomics

## Abstract

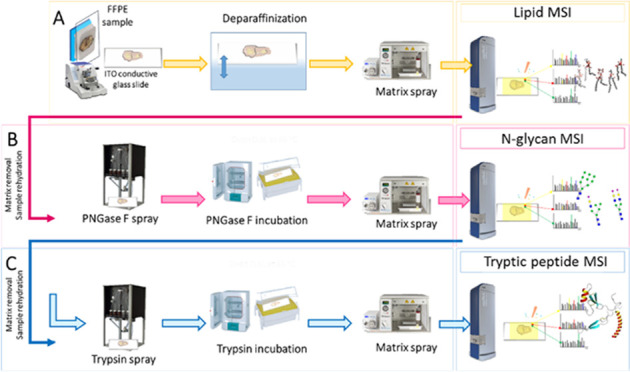

Mass spectrometry imaging (MSI) is an emerging technology that is capable of mapping various biomolecules within their native spatial context, and performing spatial multiomics on formalin-fixed paraffin-embedded (FFPE) tissues may further increase the molecular characterization of pathological states. Here we present a novel workflow which enables the sequential MSI of lipids, N-glycans, and tryptic peptides on a single FFPE tissue section and highlight the enhanced molecular characterization that is offered by combining the multiple spatial omics data sets. In murine brain and clear cell renal cell carcinoma (ccRCC) tissue, the three molecular levels provided complementary information and characterized different histological regions. Moreover, when the spatial omics data was integrated, the different histopathological regions of the ccRCC tissue could be better discriminated with respect to the imaging data set of any single omics class. Taken together, these promising findings demonstrate the capability to more comprehensively map the molecular complexity within pathological tissue.

## 1 Introduction

The field of spatial omics is rapidly evolving given that the techniques it encompasses maintain the native spatial relationship between biomolecules and the cellular network in which they are found.^1,2^ As such, they allow for a more complete overview of the complex biology which occurs in pathological tissue to be visualized, being particularly relevant in heterogeneous tumor biology.^3^ Among the spatial omics techniques available, mass spectrometry imaging (MSI) offers a powerful insight into the chemical biology of pathological tissue in a high-throughput approach where several hundreds of biomolecules can be monitored within a single experiment, in stark contrast to standard histological approaches, and it can thus help to generate a more distinct and complete molecular snapshot of the disease.^4−6^

In particular, matrix-assisted laser desorption/ionization (MALDI)-MSI) is one of the most versatile imaging techniques due to its capability to map the distribution of a wide range of biomolecules. Moreover, it also provides semiquantitative information which provides an indication regarding the relative abundances of the mapped biomolecules and thus highlight those whose regulation may be altered in different regions of pathological tissue and, for this reason, is therefore readily employed in clinical spatial omics studies.^7−10^ In these instances, clinical samples are routinely available in the form of formalin-fixed paraffin-embedded (FFPE) tissues given that they represent the gold standard for specimen preservation in pathology units.^11^ Accordingly, on-tissue trypsin digestion protocols were first developed to render spatial proteomics studies feasible,^12,13^ and these protocols have been applied to map the molecular heterogeneity in multiple diseases and correlate molecular signatures with disease type and outcome.^14,15^

However, the technique is also capable of mapping biological heterogeneity at multiple molecular levels, including the N-glycome, which was targeted given that aberrant protein glycosylation has been shown to be connected with the development of certain cancers and provides complementary information to that obtained from the proteome.^16−18^ Considering this, Heijs et al. highlighted the possibility to perform multimodal MSI on a single tissue section,^19^ mapping both N-linked glycans and proteolytic peptides utilizing a sequential on-tissue digestion protocol, and in doing so, they highlighted the potential to combine information at multiple molecular levels. However, it is apparent that the lipidome may also offer crucial insights into the pathological status of tissue, and while many lipid species are depleted from FFPE tissue by the use of paraffin wax and organic solvents during the processing of the tissue,^8^ recent studies employing MALDI-MSI^20^ and Fourier transform infrared spectroscopy^21^ have demonstrated that some of these solvent-resistant lipid species are in fact maintained in FFPE tissues and may also provide diagnostically relevant information related to lipid reprogramming.^22−24^

Given that each of these molecular classes provides a different piece of the biological jigsaw when trying to understand the complex molecular mechanisms which underpin disease, combining these multiomics data sets in a spatial context may also support the discovery of biomarkers and aid in patient subdivision.^25−28^ It is also important to consider that clinical tissue for low incidence diseases may be scarce and the presence of small cell populations or morphological features may vary even between serial sections, and thus obtaining this multiomics information from single tissue section may be highly beneficial.^19^

In this work, we describe a spatial multiomics approach which enables the sequential MALDI-MS imaging of lipids, N-glycans, and tryptic peptides on a single FFPE tissue section. In doing so, we first highlight the feasibility using murine brain tissue and demonstrate the high level of technical reproducibility for all the omics classes. Then, as a proof-of-concept, the approach was applied to four clear cell renal cell carcinoma (ccRCC) specimens to assess the ability of this spatial multiomics approach to more comprehensively characterize the tumor tissue when combining the multilevel molecular information.

## 2 Experimental Section

### 2.1 Specimen Selection

The FFPE blocks selected for this study included wild type murine brain from male BALB/C mice sacrificed at week 16 (Ethical Approval: No. 0040933/19; Autorizzazione Ministeriale No. 169/2019-PR) as well as clear cell renal cell carcinoma (ccRCC) derived from nephrectomy specimens performed for neoplasia at the University of Milano-Bicocca, San Gerardo Hospital, Monza, Italy. The appropriate Ethical Committee approved the collection of these specimens and informed consent was obtained from all participants.^14^ In order to evaluate the technical reproducibility of the method, three consecutive sections were obtained from the murine brain. Furthermore, consecutive sections were obtained also from the ccRCC samples and used for LC-MS lipidomic analysis.

### 2.2 Fixation and Cutting

Fixation time was set at 24 h following the surgical procedure, as previously described.^14^ Four micron-thick sections were cut and mounted onto conductive glasses coated with indium tin oxide (Bruker Daltonik GmbH, Bremen, Germany).^29^ Three consecutive sections were obtained from the mouse brain, and the slides were stocked at room temperature until the day of the analysis. The three technical replicates were analyzed on three separate days.

### 2.3 Sample Preparation

#### Sample Preparation for MALDI-MSI of Lipids

The slides were first placed at 60 °C for 1 h and consecutive washes in toluene (2 × 8 min) were performed before matrix application. A matrix solution containing 10 mg/mL 9-aminoacridine (9-AA) dissolved in 70% methanol was used. The obtained solution was deposited using a HTX TM-Sprayer (HTX Technologies, LLC) following the optimized parameters indicated in Table 1. Phosphorus Red, used as a calibration standard, was spotted onto the slide before the MALDI MSI analysis.

**Table 1:** HTX-TM Sprayer Parameters Utilized for Matrix Deposition in the Sequential MALDI-MSI of Lipids, N-glycans, and Tryptic Peptides

parameter	lipids	N-glycans	tryptic peptides
nozzle temperature (°C)	85	75	75
number of passes	6	4	4
flow rate (mL/min)	0.15	0.12	0.12
velocity (mm/min)	1100	1200	1200
track spacing (mm)	2	3	3
pressure (psi)	10	10	10

#### Sample Preparation for MALDI-MSI of N-glycans

Following the MALDI-MSI of lipids, 9-AA was removed from the slides and rehydration was performed with consecutive washes in 100% ethanol (1 × 3 min), 70% ethanol (1 × 3 min), and H_2_O (2 × 2 min). A citric acid antigen retrieval (CAAR) step was performed in a bath of citrate buffer (pH 5.9, 10 mM) at 97 °C for 45 min before washes in H_2_O (20 min) prior to enzyme application. PNGase PNGase F from *Elizabethkingia meningoseptica* (Sigma-Aldrich, 2 U/mL) was deposited using an iMatrixSpray (Tardo Gmbh, Subingen, Switzerland)^30^ automated spraying system following the relevant parameters indicated in Table 2. Subsequently, overnight digestion was performed in a humidity chamber at 42 °C. Finally, 5 mg/mL α-cyano-4-hydroxycinnamic acid (α-CHCA)^5^ was dissolved in a 70% acetonitrile solution with 0.1% trifluoroacetic acid and deposited using a HTX TM-Sprayer following the optimized parameters indicated in Table 1.

**Table 2:** iMatrixSpray Parameters Utilized for the Deposition of PNGase F and Trypsin, Respectively

parameter	PNGase F	trypsin
heat bed temperature (°C)	37	37
height of the needle (mm)	45	45
distance between spray lines (mm)	1.5	2
speed of movement (mm/s)	150	160
number of spray cycles	15	15
matrix density (μL/cm^2^)	1.2	1.2

#### Sample Preparation for MALDI-MSI of Tryptic Peptides

Following the MALDI-MSI of N-glycans, α-CHCA was removed from the slides and rehydration was performed as described in the previous section. Trypsin deposition (20 ng/μL) was performed using an iMatrixSpray automated spraying system following the relevant parameters indicated in Table 2. Consequently, the samples were left in a humidity chamber overnight at 40 °C. Finally, a solution containing 10 mg/mL α-CHCA in 70% acetonitrile with 0.1% trifluoroacetic acid was deposited using a HTX TM-Sprayer following the optimized parameters indicated in Table 1.

### 2.4 MALDI-MSI Parameters

All analyses were performed using a rapifleX MALDI Tissuetyper mass spectrometer (Bruker Daltonics, Bremen, Germany) equipped with a Smartbeam 3D laser operating at 10 kHz frequency. For all the samples, MALDI-MS images were acquired with the beam scan setting of 46 μm and a raster sampling of 50 μm in both *x* and *y* dimensions.

Lipid mass spectra were acquired in reflectron negative-ion mode in the *m*/*z* range of 500 to 900. External calibration was performed using red phosphorus clusters in the *m*/*z* range of 0 to 2000.^22^ The mass spectrometer voltages applied are described in a previous publication.^24^

N-Glycan and tryptic peptide mass spectra were acquired in positive-ion mode. The measurements were performed in the *m*/*z* range from 1000 to 3000 for the analysis of N-glycans and from *m*/*z* 700 to 3000 in the case of tryptic peptides. In both cases, external calibration was performed using Peptide Calibration Standard II (Bruker Daltonics, Bremen, Germany), which was spotted onto the slide before the MALDI MSI analysis. The mass spectrometer voltages employed during the N-glycan and tryptic peptide imaging acquisitions were set as following: Ion Source 1 = 20 kV, PIE = 2.58 kV, Lens = 11.7 kV, Reflector 1 = 20.84 kV, Reflector 2 = 1.085 kV, Reflector 3 = 8.75 kV. Furthermore, a small section of cortex from a ccRCC sample, containing glomeruli, was analyzed at high spatial resolution using a beam scan setting 16 μm and a raster sampling of 20 μm in both *x* and *y* dimensions.

### 2.5 LC-MS-Based Lipidomics

In order to annotate the *m*/*z* features present within the ccRCC lipid imaging data set, consecutive tissue sections were prepared as described in the lipidomic paragraph of the MALDI-MSI workflow and lipids were extracted from the tissue as previously described.^24^

An Agilent 1290 Infinity II LC, equipped with an ACQUITY UPLC BEH C18 1.7 μm, 2.1× 100 mm Column (Waters) coupled to a 6546 LC/Q-TOF system, was used for the LC-MS analysis. The gradient was set as previously described.^24^ Samples were analyzed in full scan mode and product ion mode, using a fixed collision energy of 30 eV. The MS was operating in target MS/MS mode, negative ion polarity. The lipid identifications were obtained by performing a database search using MassHunter Qualitative Analysis (Agilent) software and an Agilent Personal Compound Database and Library (PCDL). A mass tolerance of 5 ppm was set for MS while 10 ppm for MS/MS.

### 2.6 Histological Staining

Following the sequential MALDI-MS analysis, the tissue sections were washed with ethanol (70 and 100%) and the slides were stained using hematoxylin and eosin (H&E). The slides were converted to digital format by using a ScanScope CS digital scanner (Aperio, Park Center Dr., Vista, CA, USA).^10^

### 2.7 Data Processing

Data files containing the individual spectra of each entire measurement region were imported into SCiLS Lab MVS 2021c (http://scils.de/; Bremen, Germany) for spectra preprocessing. Co-registration of the digital image of the histologically stained sample was performed to annotate regions of interest (ROIs).^31^ Average spectra of the entire samples and average spectra of the ROIs were generated and exported. These average spectra were then imported into mMass (version 5.5.0, http://www.mmass.org), where peak picking was performed.^22^

Subsequently, a list of distinct *m*/*z* features associated with the three molecular classes, respectively, were generated for murine and human samples. To do so, the *m*/*z* lists obtained from mMass were elaborated as follows.

#### Curated List of Lipid Features

The list of *m*/*z* features generated in mMass were correlated with the annotations generated in Lipid Maps. A lipid identity was putatively assigned to an *m*/*z* feature if an error lower than 0.08 Da was observed between the observed and the theoretical mass. Moreover, for ccRCC samples, additional identities were assigned to those features with an observed error of ±0.08 Da between the mass measured in MALDI-MSI and the theoretical mass of the lipid species annotated by the LC-MS data alone. Those *m*/*z* signals whose identity could not be assigned were excluded from the curated list of lipid features.

#### Curated List of N-glycan Features

The list of *m*/*z* features generated from the N-glycan data set were imported in the GlycoWorkbench software. A composition analysis tool Glyco-Peakfinder^32^ was used to estimate the quantities and classes of monosaccharide components of the N-glycan structure for each *m*/*z* feature. Only sodiated adducts were considered. A putative identity was assigned if an error of ±0.05 Da was observed between the measured and theoretical mass of the N-glycan. Those *m*/*z* signals whose identity could not be assigned were excluded from the curated list of N-glycan features.

#### Curated List of Tryptic Peptides Features

The list of *m*/*z* features generated from the tryptic peptide data set of murine brain tissue was compared against an in-house library of in silico digested proteins. The library was generated performing the in silico trypsin digestion of a list of 280 murine proteins, identified in a MALDI-MSI based proteomic analysis of whole murine brain by Heijs et al.^33^ Conversely, the list of *m*/*z* features obtained from human ccRCC samples was compared to an in-house library of trypsin digested peptides that had been previously identified using a nLC-MS/MS proteomic approach from FFPE ccRCC samples.^14^ For both the murine brain and human ccRCC samples, an *m*/*z* feature was included in the curated list and assigned a putative identity if at least one tryptic peptide matched with an error of ±0.05 Da between the mass measured by MALDI-MSI and the theoretical mass present in the library.

### 2.8 Statistical Analysis

#### Evaluation of the Technical Reproducibility in Murine Brain Tissue

The absolute intensities of these *m*/*z* features included in the curated mass list of lipids, N-glycans, and tryptic peptides of murine brain tissue were used to calculate the coefficient of variance % (CV%) across the three technical replicates. Thus, a CV% for each *m*/*z* feature was obtained, and they are presented as mean and standard deviation.

#### Evaluation of the Discriminatory Capability of the Lipid, N-Glycan, and Tryptic Peptide Data Sets in ccRCC Tissue

The curated *m*/*z* feature lists obtained for the three molecular classes were imported in SCiLS Lab MVS 2021c, respectively, in order to obtain the intensity of each feature within the annotated ROIs. Three distinct data sets were organized in tables with the *m*/*z* features in columns and the different ROIs in rows. The same ROIs were selected for each of the different data sets and were grouped in four main histopathological regions: tumor, tumor infiltrating leukocytes (TILs), inflamed tissue, and leukocytes (Supplementary Figure S3). The first two macro-groups were internal to the tumoral area, while the latter two were extra-tumoral. Within the inflamed tissue, ROIs selected from the pseudocapsule and from a portion of medulla were included. Additionally, the ROIs included in the tumor group were selected both from regions containing grade 2 (G2) tumor and regions containing a tumor cells with histological signs of initial transition from G2 tumor cells to G3 (G2-G3). The H&E stained images of the additional ccRCC section analyzed are provided in Supplementary Figure S4.

Each data set was then mean normalized by row and, following this, a fourth multiomic data set was obtained by merging the lipidomic, N-glycomic, and proteomic data sets. Exploratory analysis of each data set was performed through principal component analysis (PCA), on scaled and centered data, and a molecular similarity of the regions. The HCA was carried out using the complete linkage method to identify similar clusters of data on principal components. These components were extracted from the principal component analysis (PCA) as those that explained the maximum variance of the original independent variables. Statistical analyses were performed using the open-source R software v.3.6.0 (R Foundation for Statistical Computing, Vienna, Austria).

## 3 Results

**Figure 1:**
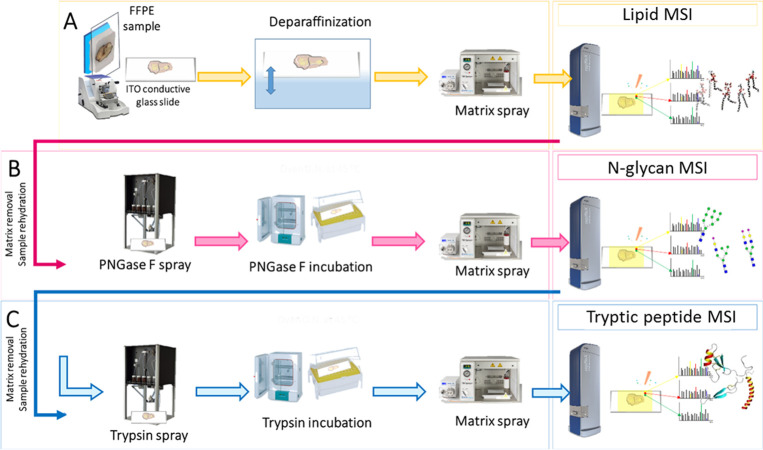
Spatial multiomics workflow using MALDI-MSI. Sample preparation for the sequential MS-imaging of lipids (A), N-glycans (B), and tryptic peptides (C) on the same FFPE tissue section.

The analytical workflow was organized in a manner that ensured that the spatial lipidomics acquisition did not interfere with the protein cross-links present in the tissue and facilitated the subsequent MSI of N-glycans, and tryptic peptides. This workflow is presented in Figure 1.

### 3.1 Technical Reproducibility Evaluated in Murine Brain Tissue

**Figure 2:**
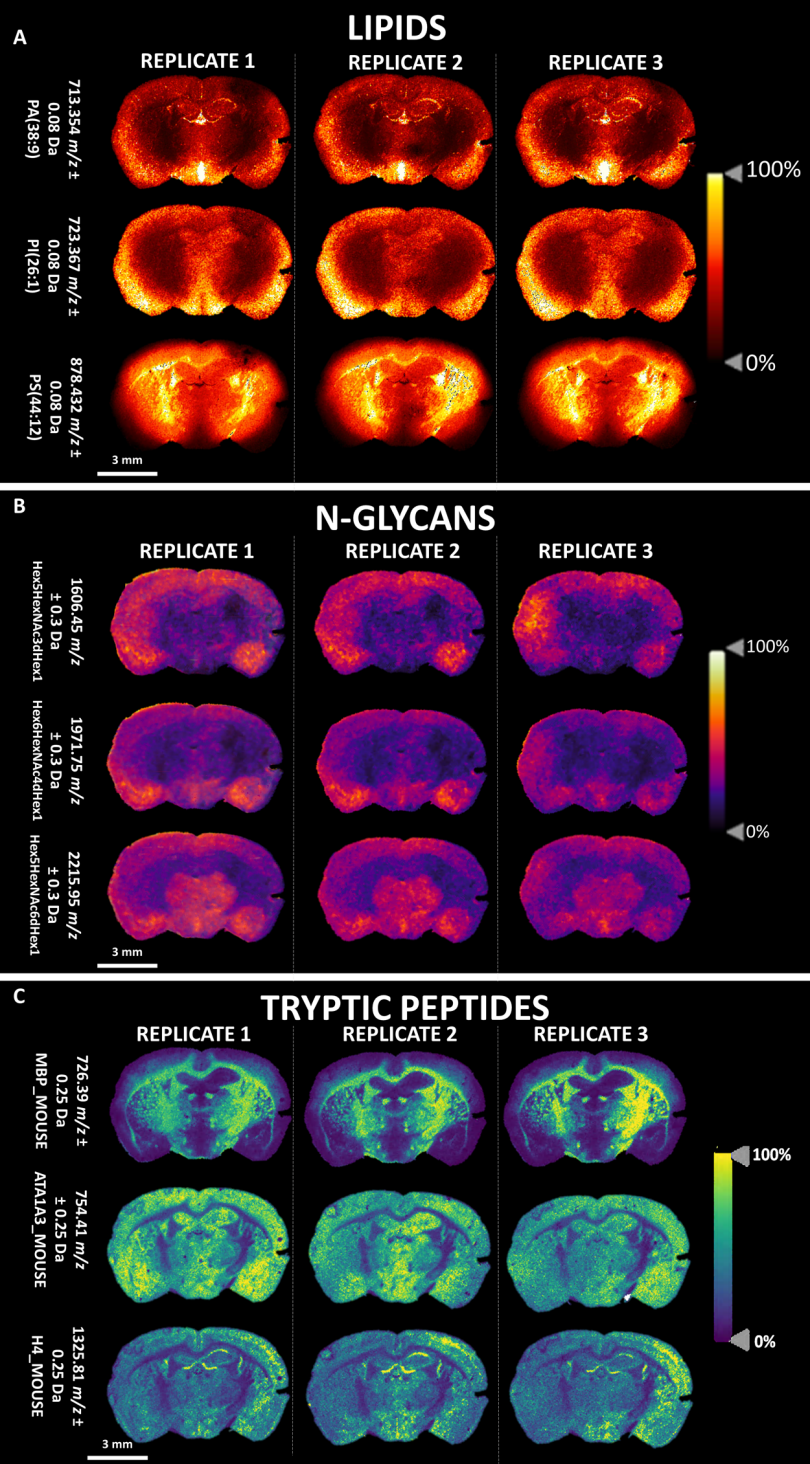
MALDI-MS images of lipids, N-glycans, and tryptic peptides in three technical replicates of murine brain tissue. The tissue distribution of the selected *m*/*z* features (rows) are highlighted in each of the technical replicates (columns), with a different color scheme for each omics class. Scale and intensity bars are provided.

Initially, the technical reproducibility of the method was evaluated in murine brain which was selected in order to decrease the biological variability between serial tissue sections. Comparing the average spectra obtained from lipid, N-glycan, and tryptic peptide imaging of the three technical replicates which were analyzed on three separate days (Supplementary Figure S1), respectively, a high degree of similarity can be observed. Accordingly, the coefficient of variance (CV%) observed for each data set was lower than 20% (lipids: 4 ± 2%; N-glycans: 11 ± 9%; tryptic peptides: 20 ± 5%). When evaluating the tissue regions highlighted by the different biomolecules, both the lipids and tryptic peptides underlined hippocampal formation, white, and gray matter, whereas the N-glycans allowed the cortex, hypothalamus, and thalamus regions to be differentiated. In particular, the highest intensity of the lipids signals putatively identified as PA(38:9) and PI(26:1), as well as the tryptic peptides of murine AT1A3 (Sodium/potassium-transporting ATPase subunit alpha-3) and H4 (Histone H4), was observed to be in the nuclei-rich regions of the gray matter. Conversely, PS(44:12) and the MBP (Myelin basic protein) specifically highlighted the white matter. Moreover, three N-glycan structures highlighted different regions of the gray matter: Hex5HexNac3dHex1 and Hex6HexNac4dHex1 for the cerebral cortex, whereas Hex5HexNac6dHex1 for the interbrain (Thalamus and Hypothalamus). The MALDI-MS images which represent the tissue distribution of these lipids, N-glycans, and tryptic peptides in the three technical replicates are presented in Figure 2.

### 3.2 Sequential MALDI-MSI of Lipids, N-glycans, and Tryptic Peptides on Clinical ccRCC Specimens

**Figure 3:**
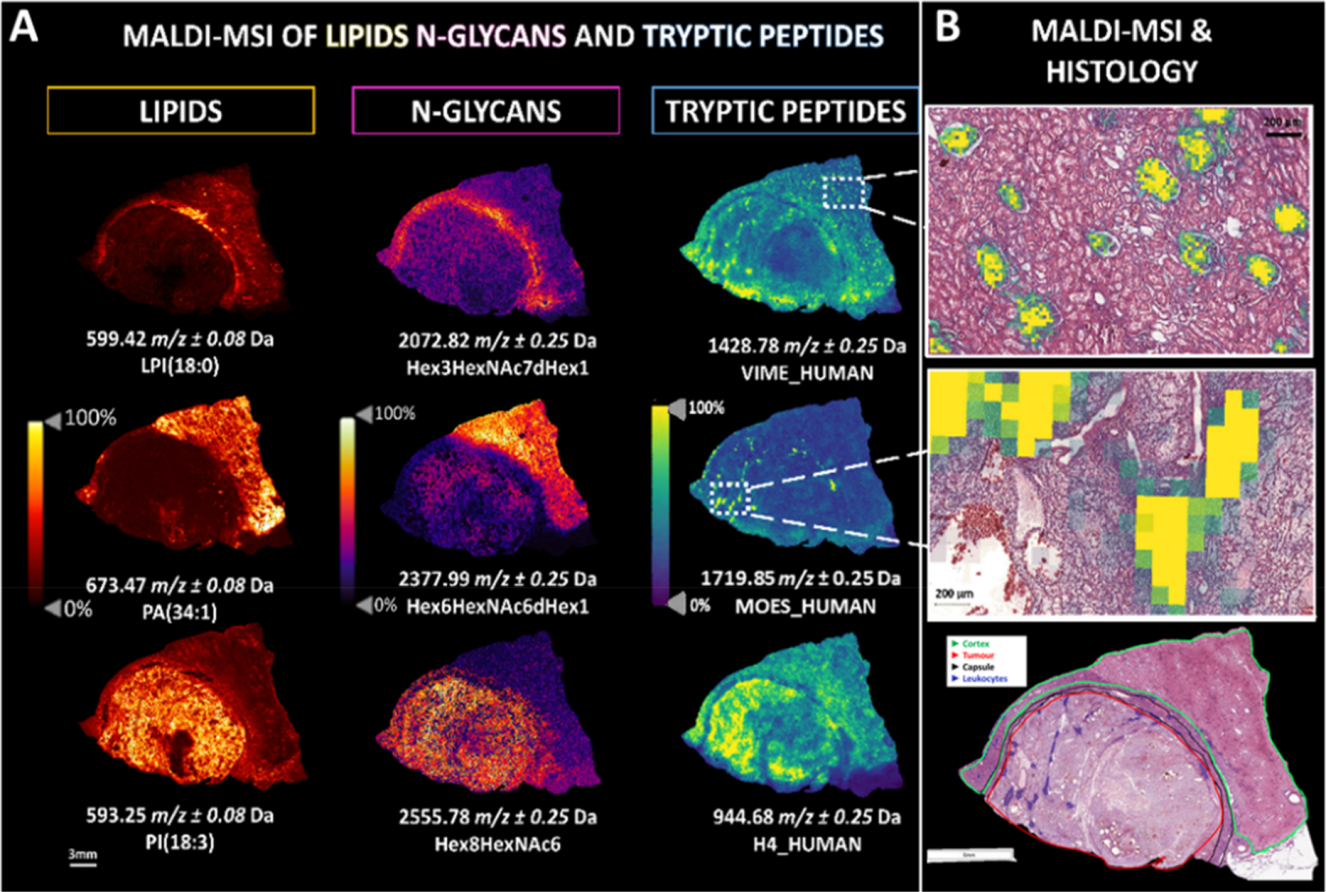
(A) Sequential MALDI-MSI of lipids, N-glycans, and tryptic peptides on the same ccRCC tissue section. The molecular annotation for each *m*/*z* feature are provided along with scale and intensity bars. (B) The insets present the tissue distribution of vimentin (*m*/*z* 1428.72) and moesin (*m*/*z* 1719.47), colocalized to the glomeruli and tumor infiltrating leukocytes (TILs), respectively.

As a proof of concept, this spatial multiomics approach was applied to clinical ccRCC specimens which were represented by more complex histopathology, whose annotations are provided in Supplementary Figures S3 and S4. Several *m*/*z* features from the lipid, N-glycans, or tryptic peptide MSI data sets, respectively, were able to highlight different regions within the tissue section (Supplementary Figure S2). In particular, the *m*/*z* features putatively identified as LPI(18:0) and Hex3HexNac7dHex1 were able to highlight the capsule of the tumor, those corresponding to PA(34:1) and Hex6HexNac6dHex1 highlighted the cortex, while PI(18:3), Hex8HexNAc6, and histone H4 highlighted the tumor region. The tissue distributions of these features are exemplified in Figure 3A.

Additionally, in order to evaluate whether the multiple tissue washes resulted in a loss of tryptic peptide localization, higher spatial resolution MALDI-MSI, using a 20 μm raster setting, was also performed on regions of nontumor tissue. Using proteins with a known tissue localization in the kidney as references, the signal for vimentin (*m*/*z* 1428.72) remained well localized to the glomeruli, as reported in Figure 3B (top). Moreover, the tryptic fragment of moesin (*m*/*z* 1719.47) was colocalized with tumor infiltrating leukocytes (TILs), as also reported in Figure 3B (bottom).

### 3.3 The Ability of Lipids, N-glycans, and Tryptic Peptides to Distinguish Discrete Histopathological Regions in ccRCC Tissue

To better demonstrate the capability of lipids, N-glycans, and tryptic peptides to better distinguish distinct histopathological regions in ccRCC tissue, descriptive statistical analyses were performed using the data sets obtained from the three molecular levels. Four main regions were taken into account: tumor tissue (grades (G) 2 and 2–3), inflamed tissue, leukocytes, and TILs. Moreover, one of the specimens also included both medulla and pseudocapsule regions of interest (ROIs). All the ROIs of inflamed tissue and leukocytes were selected from the extra-tumoral compartment (Supplementary Figure S3).

**Figure 4:**
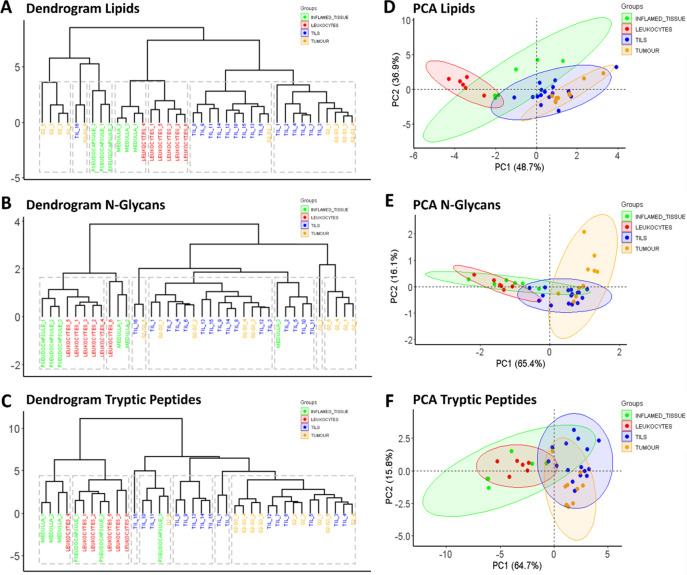
HCA dendrograms (top) and PCA score charts (bottom) generated from the lipid (A,D), N-glycan (B,E), and tryptic peptide (C,F) data sets, highlighting the characteristics of each molecular level to better distinguish different histopathological regions of ccRCC tissue. Legends are provided. 95% confidence intervals are highlighted by their respective background color.

At first glance, the hierarchical clustering (HCA) and the principal component analyses (PCA) performed on the lipids, N-glycan, and tryptic peptide data sets, respectively, clearly indicate that each molecular class was able to reveal changes within the histopathological regions at different levels. Looking at the results of each molecular data set separately, the HCA dendrogram obtained using the lipids data set (Figure 4A) separates the leukocytes and the medulla inflamed tissue from the tumor and TILs regions, under two different branches. The pseudocapsule clustered together with the intratumoral regions (TILs and tumor) but could be separated after the second and third ramification of the left-branch. These differences were also reflected in the PCA score chart where a partial overlap of the TILs and tumor regions (in blue and orange, respectively), and of the inflamed and leukocytes regions (green and red, respectively), could be observed in Figure 4D and Supplementary Figure S5A.

Second, the HCA of the N-glycan data set (Figure 4B) showed a discrete separation between the intratumoral regions and the external ones, apart from one of the medulla regions. The second division enabled the tumor regions containing exclusively G2 tumor cells to be separated from the rest of the tumor regions and the TILs. This was also observed in the PCA score chart (Figure 4E), where it was observed that the five tumor regions did not overlap with the region of TILs belonging to the G2 tumor. Moreover, a partial overlap between the inflamed tissue and the leukocyte-rich regions was observed (Figure 4E and Supplementary Figure S5B), in accordance with the level of separation observed in the dendrogram.

Finally, the HCA and the PCA score chart obtained from the tryptic peptide data set (Figures 4C and 4F, respectively) provided additional information. The first division in the dendrogram separated extra-tumoral regions from the intratumoral ones, except for one inflamed tissue region. Under the intratumoral group, a partial separation of G2-G3 tumor regions from G2 and TILs could be highlighted. This was reflected in the PCA score chart (Figure 4F) where, at first glance, a partial overlap of the tumor regions and TILs was observed. Moreover, the PCA showed a different direction of the intra- and extra-tumoral regions along the components, with a stronger separation of these two groups highlighted in Supplementary Figure S5C.

### 3.4 Integration of the Spatial Multiomics Data Sets for the Enhanced Molecular Distinction of ccRCC Tissue

**Figure 5:**
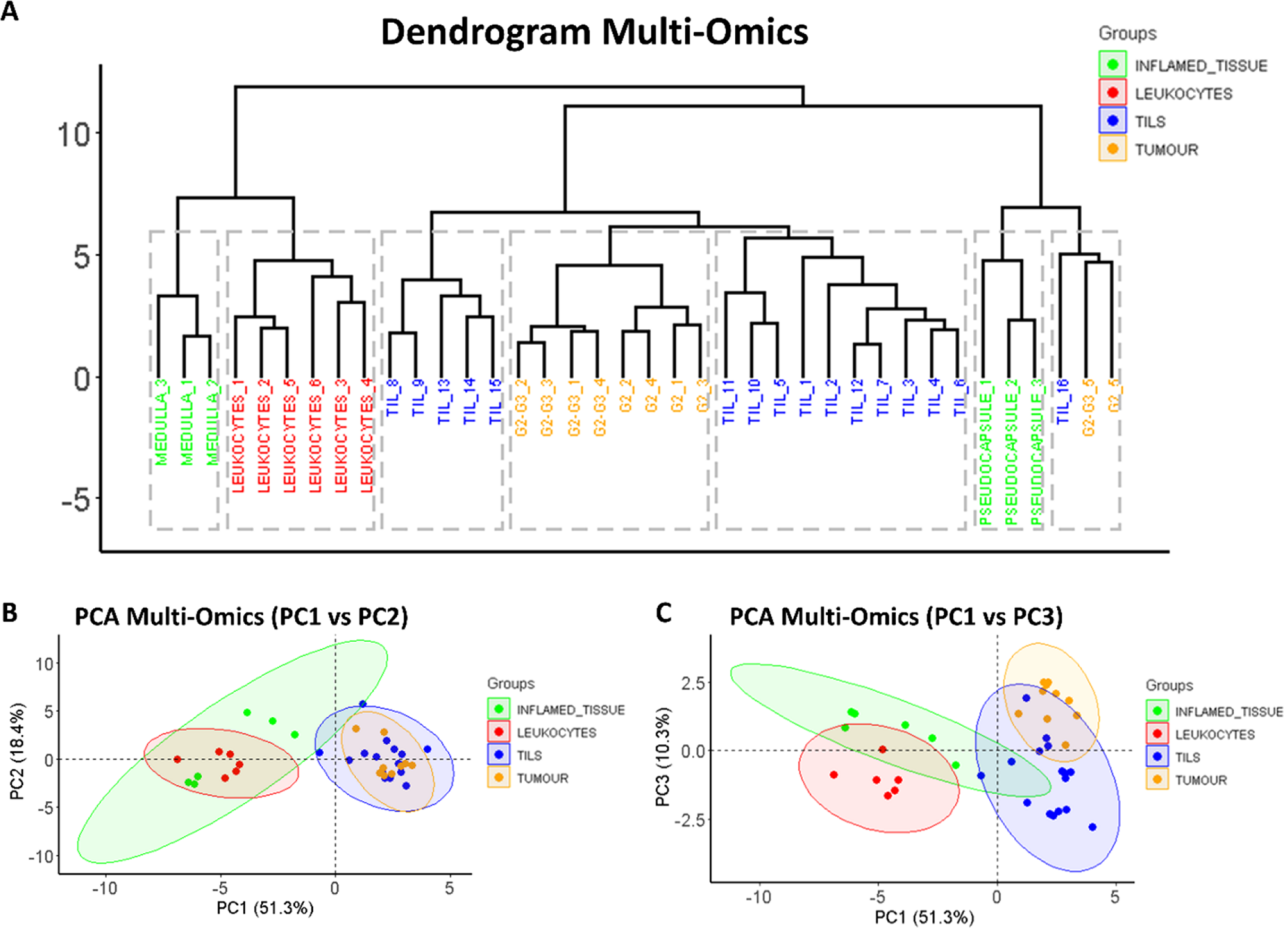
HCA dendrogram (A) and principal component analysis score charts (B,C) of the integrated spatial multiomics data set comprehensive of lipid, N-glycan, and tryptic peptide information. B represents PC1 (*x* axis) vs PC2 (*y* axis), while C represents PC1 (*x* axis) vs PC3 (*y* axis), which increased the ability to distinguish among the four major histopathological groups. 95% confidence intervals are highlighted by the respective background color.

The qualitative statistical analysis of the integrated multiomics data set was performed considering the same ROIs that were previously described. Accordingly, Figure 5 presents the results obtained from the HCA and PCA of the integrated spatial omics data. Foremost, the first ramification in the HCA dendrogram separated the medulla and the leukocyte regions from those remaining (Figure 5A). The pseudocapsule regions, even if part of the inflamed macro-group, were now clustered together and separated from the remaining intratumoral regions. However, the HCA showed a clear clustering of the three extra-tumoral subtypes (pseudocapsule, medulla, and leukocytes). Focusing on the intratumoral portion, separation between TILs and the tumor regions is evident. Moreover, HCA could depict a further separation among tumor regions; the G2-G3 regions were clustered together and separated from those corresponding with the G2 tumor cells, except for two tumor regions. This degree of separation was not possible using the individual molecular classes (Figure 4). Finally, the multiomics data set was able to underline a further separation among the TILs group.

These results were also confirmed within the PCA score chart. First, a distinct separation among the extra-tumoral and intratumoral regions was observed along PC1 and PC2 (Figure 3B). Additionally, the inflamed tissue regions (green) showed the largest confidence interval (95% CI) between the four macro-groups. This could be explained by the separation of the medulla and pseudocapsule regions along the second component, which was also observed in the HCA dendrogram. Moreover, while the tumor and TIL regions partially overlapped when considering PC1 and PC2 (Figure 5B), these could then be separated when considering PC1 and PC3 (Figure 5C). In fact, in this instance, considering PC3 also increased the ability to distinguish among the four major histopathological groups and also highlighted further molecular heterogeneity among the TILs annotated (Figure 5C), as demonstrated by the large 95% CI, and supports the observations noted in the HCA. Interestingly, the features which contributed most greatly to the separation observed along principal components one to three of the multiomics data set also included a combination of lipids, N-glycans, and tryptic peptides (Supplementary Figure S6).

## 4 Discussion

The “omics era” has opened advanced frontiers in our search for novel disease biomarkers and to understand the molecular basis of pathologies. However, spatial context is also highly relevant to this cause, and techniques which are able to maintain the spatial relationship of those molecules within a complex cellular network are becoming ever more important. Moreover, the more recent “multi-omics” neologism indicates a modeling biological approach that uses analytical approaches, which can target the diverse omics levels, to describe biological systems.^25,34,35^ However, it is well recognized that each molecular class contributes differently to the molecular description of the biological system under investigation,^36,37^ and combining this multiomic information, within its native spatial context, may thus provide a more complete understanding of various pathologies.^38−40^

With regards to the multiomics prospects of mass spectrometry imaging, the possibility to perform sequential MALDI-MSI analysis of N-glycans and tryptic peptides on a single FFPE section was recently described,^19^ however, this study did not evaluate the potential of integrating the multimodal MSI data set. Moreover, findings from the spatial lipidome may provide further relevant information to the molecular jigsaw and recent studies published by our group demonstrated the possibility to perform spatial lipidomics using MALDI-MSI on these type of samples,^22−24^ highlighting molecular alterations which correlated with the histopathological and immunohistochemical landscape of the tissue. Furthermore, to appreciate molecular changes in small cellular groups that could be lost between consecutive sections, it is highly beneficial to perform this spatial multiomics sequence on the same tissue section.^41^ This is also relevant when studying rare diseases, or those deriving from small tissue biopsies, where the quantity of clinical tissue is scarce.

Here, we describe a spatial multiomics approach using MALDI-MSI which enables the sequential analysis of lipids, N-glycans, and tryptic peptides on a single FFPE tissue section (Figure 2). First, the technical reproducibility of the method was evaluated on consecutive tissue sections of murine brain tissue, which was selected in order to decrease the biological variability between serial tissue sections. As expected, the lowest CV% was observed in the lipids MALDI-MSI data set, which was to be expected given that limited number of analytical steps performed prior to this point. However, the CV% was 11 ± 9% and 20 ± 5% for the N-glycan and tryptic peptide data sets, respectively. Naturally, a larger degree of variability was observed with respect to that observed in the lipid MALDI-MSI data set, which is to be expected considering the additional sources of possible analytical variability, such as the antigen retrieval step and enzymatic digestion. Nevertheless, the mean CV% obtained from each of the sequential MALDI-MSI analysis lay within a range comparable to the CV threshold of 20% recommended by the European Medicine Agency (EMA) for analytical techniques.^42^

When the spatial distribution of the detected lipids, N-glycans, and tryptic peptides was evaluated, it soon became apparent that each molecular class showed tendencies to better underline different regions of the brain tissue, indicating their complementary nature (Figure 1). This was also consistent among the three technical replicates. Interestingly, both the lipids and tryptic peptides well underlined the white matter of the brain, with PS(44:12) and a tryptic fragment of the MBP being colocalized to this region. This also supports that these findings are considered with the known biochemistry of the brain tissue, considering that there is a known interaction between the myelin basic proteins and phosphatidylserines.^43^

Subsequently, the potentiality of this spatial multiomics approach in pathological tissue was assessed, using ccRCC specimens as proof-of-concept. A histological overview of the four different ccRCC clinical specimens are provided in Supplementary Figure S2. As already observed in the murine brain tissue, each of the molecular levels again showed tendencies to better underline different histopathological regions of the ccRCC tissue (Figure 3). Moreover, the spatial distribution of the bioanalytes, including that of the tryptic peptides (Figure 3B), did not appear to be significantly impacted by the multiple washes and sequential treatments to which the tissue was subjected and was compatible with the 20 μm lateral resolution employed for this evaluation. For example, a tryptic fragment relating to vimentin (*m*/*z* 1428.72), a protein constitutively expressed in podocytes at high levels and used as a glomerular marker in immunohistochemistry (IHC),^44^ in fact remained well localized to the glomeruli of the kidney. The same localization was also previously demonstrated by MALDI-MSI in renal tissue that had undergone the in situ tissue digestion process alone.^45^ Similar results were also observed for further proteins, such as Hemoglobin Subunit A (*m*/*z* 1071.54), which remained well localized to tissue regions with evident hemorrhaging, as highlighted in Supplementary Figures S2–S4. Moreover, a tryptic fragment related to moesin (*m*/*z* 1719.47) was found to be colocalized and in high relative abundance in regions of TILs. This further confirms that the native tissue distribution of the tryptic peptides has been minimally impacted but, furthermore, may be of particular interest in future clinical studies given the proven role of this protein in enhancing the lymphocyte infiltration^46^ and, to the best of our knowledge, has not been readily detected using this technology. Despite this promise, considering that multiple washing and enzymatic digestion steps are required, a certain degree of analyte delocalization cannot be ruled out and could be considered a possible limitation of this method, especially if a single-cell spatial resolution wishes to be achieved.

However, considering that multiple washing and enzymatic digestion steps were performed, a certain degree of analyte delocalization may be expected and could be considered a possible limitation of this method, especially if a single-cell resolution wishes to be achieved.

To confirm the capability of each molecular class to distinguish among the different histopathological regions of ccRCC tissue, qualitative statistical analyses were performed. In support of what was observed in the MALDI-MS images themselves, each data set led to a complementary separation of the histopathological regions (Figure 3). More precisely, the spatial lipidome was particularly adept to separating leukocytes from the rest of the tissue (Figure 4A and 4D), supporting what was previously observed by our group.^24^ Conversely, the N-glycans facilitated a discrete separation between the intratumoral regions and nontumor tissue (Figure 4B and 4E), in line with what one could expect considering tumor biology,^17^ while the tryptic peptides were more adapted to distinguishing the inflammatory environment around the tumor as well as the capsule itself.^47^

When these individual data sets were then combined into one large multiomics data set, the histopathological regions were separated with greater power (Figure 5) and, in fact, could all easily be distinguished from one another. This was not possible using the lipid, N-glycan, or tryptic peptide data sets in isolation. Moreover, greater molecular heterogeneity was also uncovered among the TILs (Figure 5C and 5F), which would suggest that diverse subpopulations of leukocytes^48^ were able to be detected, although immunohistochemical confirmation is required.

While this study focuses on known histopathological regions as proof of concept, it underlines the increased molecular coverage that is obtained by using a spatial multiomics approach and can lead to a more comprehensive characterization of diseased tissue. This powerful approach has the potential to be exploited in clinical studies where the multiomics data can be integrated to aid the discovery of new subgroups of patients with different molecular features and possible different outcomes.^49^ Conversely, these findings also encourage the further development of more powerful bioinformatics tools which can be used to mine these spatial multiomic data sets and uncover hidden molecular patterns which arise as a result of the relationship between these multiple molecular levels.^50^

Taken together, the spatial-multiomics approach presented here provides the ability to map the spatial distribution of lipids, N-glycans, and tryptic peptides on a single FFPE tissue section. As demonstrated in ccRCC tissue, the different molecular levels are able to provide complementary information which, when coupled with more extensive molecule identification, may contribute to our biological understanding of pathologies. Moreover, the integration of this multiomic data enhances the molecular characterization of tissue and shows great promise for application in clinical studies.

## 5 Conclusions

In this work, we present a novel MALDI-MSI protocol which enables the spatial multiomics analysis of lipids, N-glycans, and tryptic peptides on a single FFPE tissue section. Therefore, we first investigated the feasibility on mouse brain sections and demonstrate a high level of reproducibility for all the analytes in technical replicates. Moreover, when this method was applied to heterogeneous ccRCC samples, each molecular level was able to distinguish diverse histopathological features. Additionally, the qualitative statistical analyses demonstrated that integrating the three molecular data sets could improve the separation across distinct regions of interest of a ccRCC section.

Future improvements of the methodology will allow one to obtain multimolecular information with a greater lateral resolution and a greater mass resolution. Finally, the promising data obtained encourage the use of this multiomic MALDI-MSI method on a larger cohort of ccRCC samples to assist future clinical studies on archived samples.

## Notes

The data set associated with this paper was uploaded to Massive on September 29, 2022 and assigned an identifier of 78a0482cdfe34a9e9e422c6eabff5b56.

## Supporting Information Available

Supplementary Figure S1: Average MALDI mass spectra of lipids, N-glycans, and tryptic peptides in murine brain tissue; Supplementary Figure S2: Spatial multiomics of lipids, N-glycans, and tryptic peptides on four different ccRCC tissue sections; Supplementary Figure S3: Hematoxylin and eosin staining of the ccRCC section used for the statistical analysis; Supplementary Figure S4: H&E stained images of the additional ccRCC section used; Supplementary Figure S5: PCA score charts generated from the lipid, N-glycan, and tryptic peptide data sets; Supplementary Figure S6: The top ten contributing variables (features) to the separation observed along the first three components of the PCA analysis for the lipid, N-glycan, tryptic peptide, and multiomic data sets; Supplementary Table S1: List of *m*/*z* features included in the curated mass list, identified by liquid chromatography coupled with tandem mass spectrometry (LC-MS/MS) following extraction from ccRCC tissue (PDF)
