# Correction to Spatial Multiomics of Lipids, N-Glycans, and Tryptic Peptides on a Single FFPE Tissue Section

**DOI:** 10.1021/acs.jproteome.3c00217

**Published:** 2023-05-05

**Authors:** Vanna Denti, Giulia Capitoli, Isabella Piga, Francesca Clerici, Lisa Pagani, Lucrezia Criscuolo, Greta Bindi, Lucrezia Principi, Clizia Chinello, Giuseppe Paglia, Fulvio Magni, Andrew Smith

This paper was originally not requested by the authors for consideration in the submissions for the Methods for Omics Research 2023 Special Issue, https://pubs.acs.org/toc/jprobs/22/5. However, Guest Editors of this Special Issue later identified this paper as a contribution worth mention related to the Special Issue. Therefore, the paper has been linked to the Special Issue.

