# Supplementary material for: Spatial Multiomics of Lipids, N-Glycans, and Tryptic Peptides on a Single FFPE Tissue Section
Source: J Proteome Res. 2022 Oct 19;21(11):2798–809. doi: 10.1021/acs.jproteome.2c00601 (PMC9639202; doi:10.1021/acs.jproteome.2c00601)
Supplement: Supplementary file 1 — pr2c00601_si_001.pdf [file pr2c00601_si_001.pdf]

Spatial multi-omics of lipids, N-glycans, and tryptic peptides on a single FFPE tissue section

Vanna Denti[1], Giulia Capitoli[2], Isabella Piga[1], Francesca Clerici[1], Lisa Pagani[1], Lucrezia Criscuolo[1], Greta Bindi[1],

Lucrezia Principi[1], Clizia Chinello[1], Giuseppe Paglia[1], Fulvio Magni[1], and Andrew Smith[1*]

[1] Department of Medicine and Surgery, Proteomics and Metabolomics Unit, University of Milano-Bicocca, Vedano al Lambro, 20854, Italy

[2] Bicocca Bioinformatics Biostatistics and Bioimaging B4 Center, School of Medicine and Surgery, University of Milano-Bicocca, 20900 Monza, Italy

Supplementary Figure 1.

Average MALDI mass spectra of lipids, N-glycans, and tryptic peptides in murine brain tissue. The number of peaks per spectrum (p) is indicated. On the bottom, the number of common peaks for each analyte class is reported.

Supplementary Figure 2.

Spatial multi-omics of lipids, N-glycans, and tryptic peptides on four different ccRCC tissue sections. The spatial distribution and the relative intensity of four example bioanalytes for each molecular class is highlighted. Regarding the lipids, the *m/z* of the [M-H] ions related to PI(18:0), PE(22:4), PA(18:1/18:0), and PA(16:0/20:4) are reported. Regarding the N-glycans, the *m/z* of the [M+Na] ions related to Hex8HexNAc6, Hex6HexNAc4, Hex6HexNAc5, and Hex9HexNAc2 are reported. Regarding the tryptic peptides, the *m/z* of the [M+H] ions of related to MOES (Moesin), TGF1 (Transforming growth factor 1), HBA (Haemoglobin subunit alpha), and ANXA2 (Annexin A2) are reported. In particular, the tissue distribution of these features serves to highlight the capacity of the different molecular classes to underline the distinct histopathological regions.

Supplementary Figure 3.

Haematoxylin and Eosin staining of the ccRCC section used for the statistical analysis. The regions of interest (ROIs) considered in the analysis are highlighted: Pseudocapsule; medulla; leukocytes; TILs (tumour infiltrating leukocytes); G2-G3; G2. Pseudocapsule and medulla were included in the “inflamed tissue” region. G2-G3 and G2 were included in the “Tumour” region.

Supplementary Figure 4.

H&E stained images of the additional ccRCC section used. The histological annotations are reported: T= tumour; H=haemorrhagic tissue; C= Cortex; V=vessels; M= Medulla; P=pseudocapsule. The name of the sample is reported on the top.

Supplementary Figure 5.

PCA score charts generated from the lipid (A), N-glycan (B), and tryptic peptide (C) datasets, plotting PC1 (x axis) vs PC3 (y axis). A legend with the four major histopathological groups is provided (D). 95% confidence intervals are highlighted by their respective background colour.

Supplementary Figure 6.

The top ten contributing variables (features) to the separation observed along the first three components of the PCA analysis for the lipid (A), N-glycan (B), tryptic peptide (C), and multi-omic datasets (D). The dashed red line indicates a one percent contribution level.

Supplementary Table 1.

List of *m/z* features included in the curated mass list, identified by liquid chromatography coupled with tandem mass spectrometry (LC-MS/MS) following extraction from ccRCC tissue. Accurate mass, ID, and retention time (in minutes) for each feature are reported.

Supplementary Figures

Supplementary Figure 1.

Average MALDI mass spectra of lipids, N-glycans, and tryptic peptides in murine brain tissue. The number of peaks per spectrum (p) is indicated. On the bottom, the number of common peaks for each analyte class is reported.

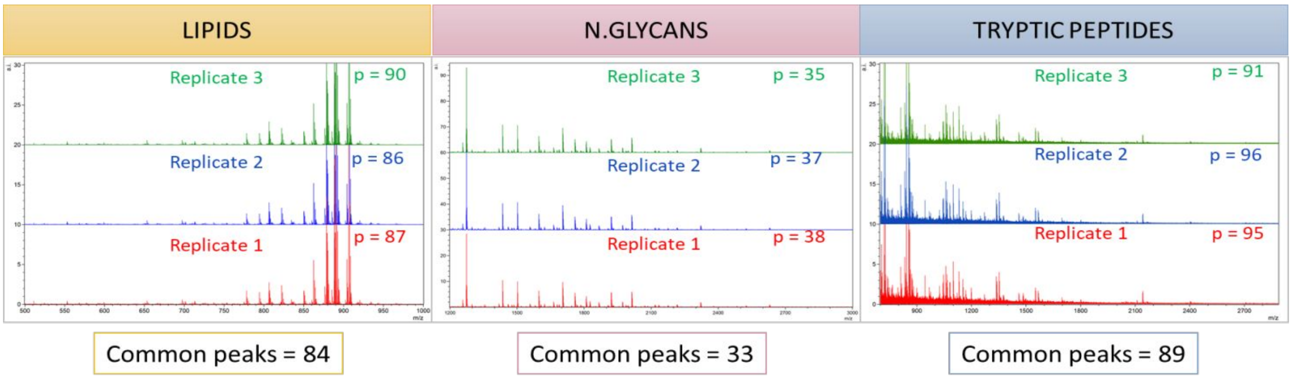

Supplementary Figure 2.

Spatial multi-omics of lipids, N-glycans, and tryptic peptides on four different ccRCC tissue sections. The spatial distribution and the relative intensity of four example bioanalytes for each molecular class is highlighted. Regarding the lipids, the *m/z* of the [M-H] ions related to PI(18:0), PE(22:4), PA(18:1/18:0), and PA(16:0/20:4) are reported. Regarding the N-glycans, the *m/z* of the [M+Na] ions related to Hex8HexNAc6, Hex6HexNAc4, Hex6HexNAc5, and Hex9HexNAc2 are reported. Regarding the tryptic peptides, the *m/z* of the [M+H] ions of related to MOES (Moesin), TGF1 (Transforming growth factor 1), HBA (Haemoglobin subunit alpha), and ANXA2 (Annexin A2) are reported. In particular, the tissue distribution of these features serves to highlight the capacity of the different molecular classes to underline the distinct histopathological regions.

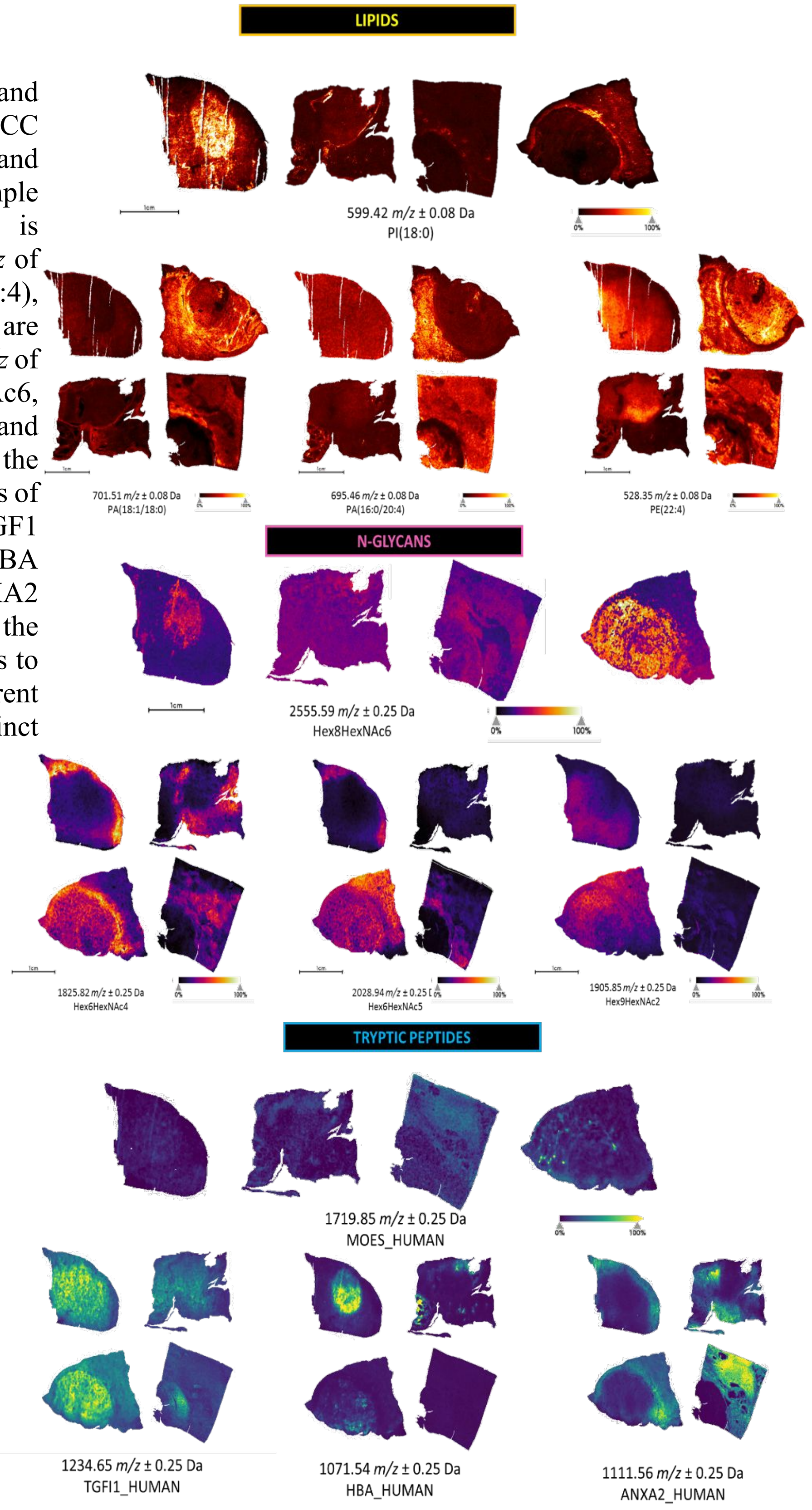

Supplementary Figure 3.

Haematoxylin and Eosin staining of the ccRCC section used for the statistical analysis. The regions of interest (ROIs) considered in the analysis are highlighted: Pseudocapsule; medulla; leukocytes; TILs (tumour infiltrating leukocytes); G2-G3; G2. Pseudocapsule and medulla were included in the "inflamed tissue" region. G2-G3 and G2 were included in the "Tumour" region.

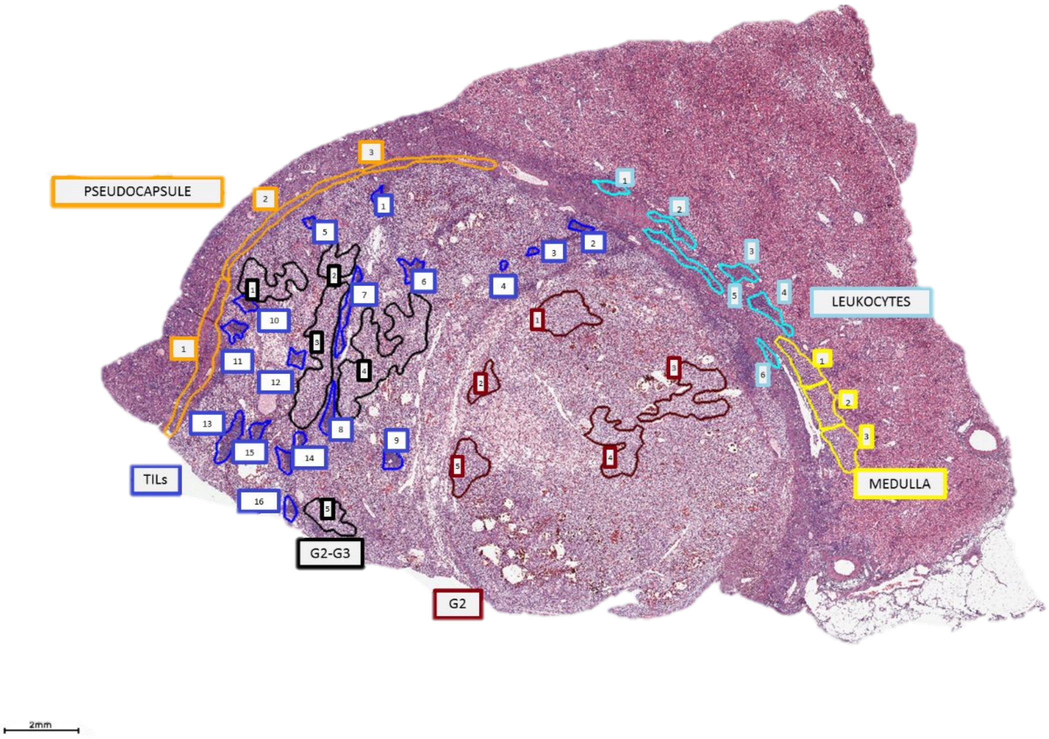

Supplementary Figure 4.

H&E stained images of the additional ccRCC section used. The histological annotations are reported: T= tumour; H=haemorrhagic tissue; C= Cortex; V=vessels; M= Medulla; P=pseudocapsule. The name of the sample is reported on the top.

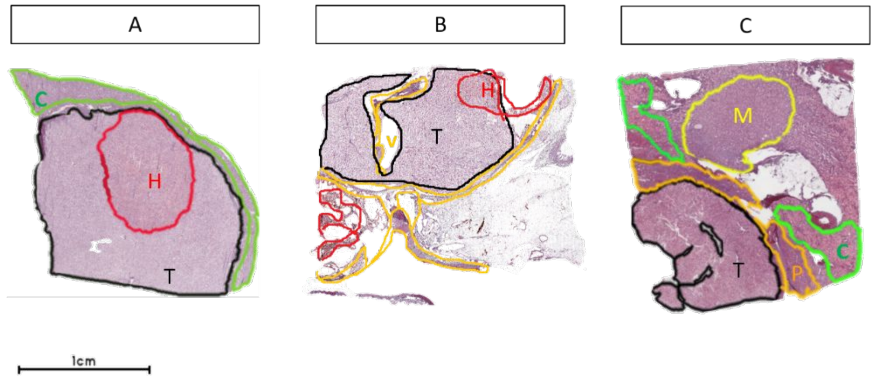

Supplementary Figure 5.

PCA score charts generated from the lipid (A), N-glycan (B), and tryptic peptide (C) datasets, plotting PC1 (x axis) vs PC3 (y axis). A legend with the four major histopathological groups is provided (D). 95% confidence intervals are highlighted by their respective background colour.

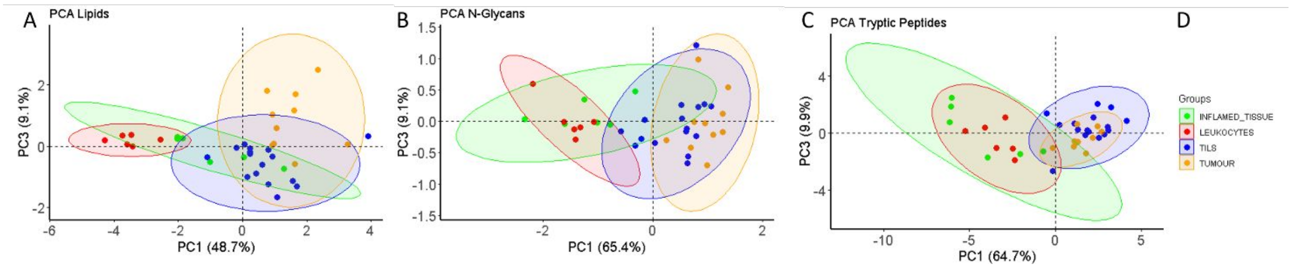

Supplementary Figure 6.

The top ten contributing variables (features) to the separation observed along the first three components of the PCA analysis for the lipid (A), N-glycan (B), tryptic peptide (C), and multi-omic datasets (D). The dashed red line indicates a one percent contribution level.

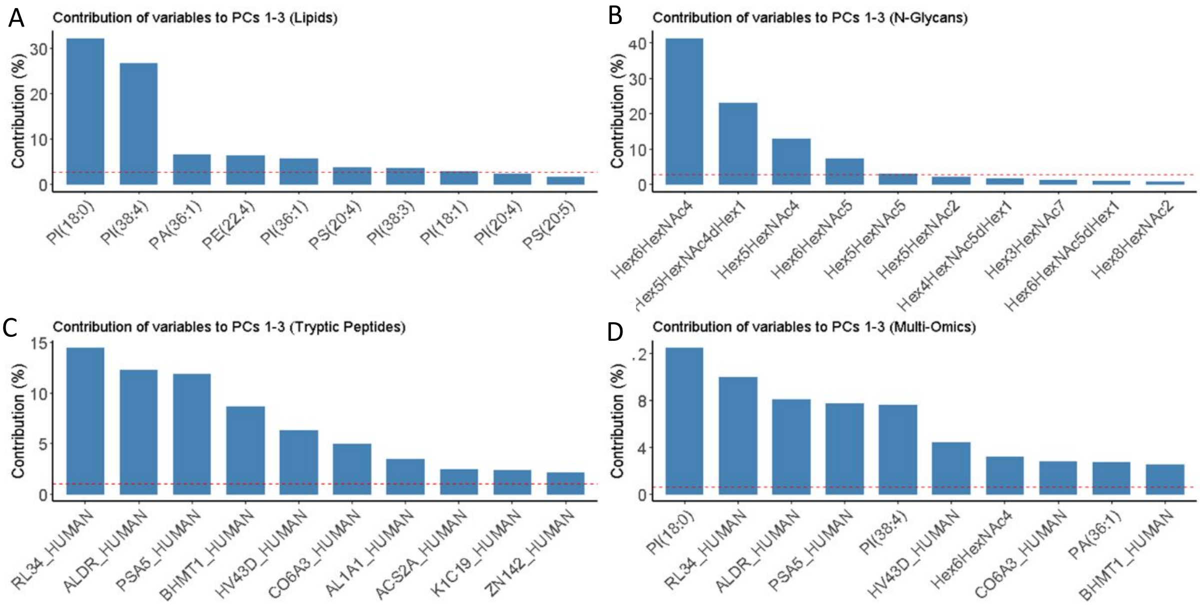

Supplementary Tables

Supplementary Table 1.

List of *m/z* features included in the curated mass list, identified by liquid chromatography coupled with tandem mass spectrometry (LC-MS/MS) following extraction from ccRCC tissue. Accurate mass, ID, and retention time (in minutes) for each feature are reported.

m/z	***ID***	**RT**
**571.2873**	LPI(16:0)	1.9
**597.303**	LPI(18:1)	1.9
**599.3189**	LPI(18.0)	2.4
**619.2869**	LPI(20:4)	1.5
**673.4793**	PA(16:0/18:1)	5.9
**695.464**	PA(16:0/20:4)	5.4
**699.4954**	PA(18:2/18:0)	6.0
**701.5108**	PA(18:1/18:0)	6.4
**723.4952**	PA(20:4/18:0)	5.9
**725.5098**	PA(18:0/18:3)	6.1
**809.5166**	PI(16:0/16:0)	5.5
**833.5158**	PI(16:0/16:0)	5.2
**835.5318**	PI(18:2/16:0)	5.5
**837.5464**	PI(16:0/18:0)	6.0
**857.5161**	PI(20:4/16:0)	5.1
**859.5315**	PI(18:2/18:1)	5.2
**861.5476**	PI(18:0/18:0)	5.6
**863.5626**	PI(18:0/18:1)	6.0
**883.5316**	PI(20:4/18:1)	5.1
**885.5477**	PI(20:4/18:0)	5.6
